# Homological scaffolds of brain functional networks

**DOI:** 10.1098/rsif.2014.0873

**Published:** 2014-12-06

**Authors:** G. Petri, P. Expert, F. Turkheimer, R. Carhart-Harris, D. Nutt, P. J. Hellyer, F. Vaccarino

**Affiliations:** 1 ISI Foundation, Via Alassio 11/c, 10126 Torino, Italy; 2 Centre for Neuroimaging Sciences, Institute of Psychiatry, Kings College London, De Crespigny Park, London SE5 8AF, UK; 3 Centre for Neuropsychopharmacology, Imperial College London, London W12 0NN, UK; 4 Computational, Cognitive and Clinical Neuroimaging Laboratory, Division of Brain Sciences, Imperial College London, London W12 0NN, UK; 5 Dipartimento di Scienze Matematiche, Politecnico di Torino, C.so Duca degli Abruzzi no 24, Torino 10129, Italy

**Keywords:** brain functional networks, fMRI, persistent homology, psilocybin

## Abstract

Networks, as efficient representations of complex systems, have appealed to scientists for a long time and now permeate many areas of science, including neuroimaging (Bullmore and Sporns 2009 *Nat. Rev. Neurosci.* **10**, 186–198. (doi:10.1038/nrn2618)). Traditionally, the structure of complex networks has been studied through their statistical properties and metrics concerned with node and link properties, e.g. degree-distribution, node centrality and modularity. Here, we study the characteristics of functional brain networks at the mesoscopic level from a novel perspective that highlights the role of inhomogeneities in the fabric of functional connections. This can be done by focusing on the features of a set of topological objects—*homological cycles*—associated with the weighted functional network. We leverage the detected topological information to define the *homological scaffolds*, a new set of objects designed to represent compactly the homological features of the correlation network and simultaneously make their homological properties amenable to networks theoretical methods. As a proof of principle, we apply these tools to compare resting-state functional brain activity in 15 healthy volunteers after intravenous infusion of placebo and psilocybin—the main psychoactive component of magic mushrooms. The results show that the homological structure of the brain's functional patterns undergoes a dramatic change post-psilocybin, characterized by the appearance of many transient structures of low stability and of a small number of persistent ones that are not observed in the case of placebo.

## 1. Motivation

The understanding of global brain organization and its large-scale integration remains a challenge for modern neurosciences. Network theory is an elegant framework to approach these questions, thanks to its simplicity and versatility [1]. Indeed, in recent years, networks have become a prominent tool to analyse and understand neuroimaging data coming from very diverse sources, such as functional magnetic resonance imaging (fMRI), electroencephalography and magnetoencephalography [2,3], also showing potential for clinical applications [4,5].

A natural way of approaching these datasets is to devise a measure of dynamical similarity between the microscopic constituents and interpret it as the strength of the link between those elements. In the case of brain functional activity, this often implies the use of similarity measures such as (partial) correlations or coherence [6–8], which generally yield fully connected, weighted and possibly signed adjacency matrices. Despite the fact that most network metrics can be extended to the weighted case [9–13], the combined effect of complete connectedness and edge weights makes the interpretation of functional networks significantly harder and motivates the widespread use of *ad hoc* thresholding methods [7,14–18]. However, neglecting weak links incurs the dangers of a trade-off between information completeness and clarity. In fact, it risks overlooking the role that weak links might have, as shown for example in the cases of resting-state dynamics [19,20], cognitive control [21] and correlated network states [22].

In order to overcome these limits, Rubinov & Sporn [13,23,24] recently introduced a set of generalized network and community metrics for functional networks that among others were used to uncover the contrasting dynamics underlying recollection [25] and the physiology of functional hubs [26].

In this paper, we present an alternative route to the analysis of brain functional networks. We focus on the combined structure of connections and weights as captured by the homology of the network. A summary of all the keywords and concepts introduced in this paper can be found in table 1.

**Table 1.** List of notations.

name	symbol	definition
graph	*G*	a graph *G* = (*V*, *E*) is a representation of a set *V* of nodes *i* interconnected by edges or links 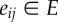 ; this interaction can be weighted, directional and signed
clique	*c*	a completely connected subgraph *C* = (*V’*, *E’*) contained in an undirected and unweighted graph *G* = (*V*, *E*) ( 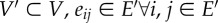 )
*k*-simplex	*σ_k_*	formally, a convex hull of *k* + 1 nodes [*p*_0_, *p*_1_, … ,*p_k_*], it is used here as a generalization to higher dimensions of the concept of link, e.g. a 2-simplex is a triangle, a 3-simplex a tetrahedron. The faces *f* of *σ_k_* are obtained as subsets of [*p*_0_, *p*_1_*,* … ,*pk*]
simplicial complex	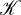	a topological space composed by attaching simplices *σ*, with two conditions: (i) if 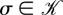 then all its faces 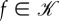 and (ii) the intersection of any two 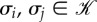 is empty or a face of both *σ_i_* and *σ_j_*.
clique complex	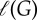	a simplicial complex built from a graph *G* by promoting every *k*-clique 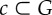 to a (*k* − 1)-simplex defined by the nodes of *c*, e.g. a 3-clique becomes a 2-simplex (a full triangle)
*k*th homology group	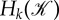	a group describing the holes of a simplicial complex 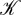 bounded by *k*-dimensional boundaries, e.g. *H*_1_ describes two-dimensional cycles bounded by 1-simplices, *H*_2_ describes three-dimensional voids bounded by 2-simplices, etc.
*H_k_* generator	*g*	an element of the generating set of *H_k_*, a subset of *H_k_* such that all elements can be expressed as combination of generators
homological scaffold	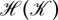	a weighted graph constructed from the persistent homology generators of *H*_1_ of a simplicial complex 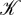

## 2. From networks to topological spaces and homology

**Figure 1.**
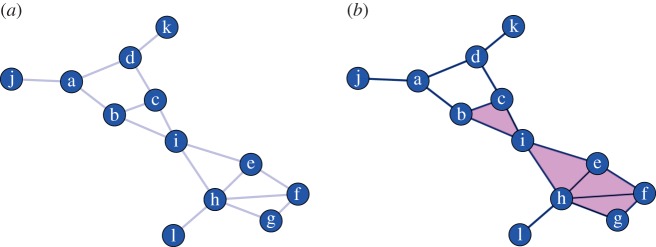
Panels (*a,b*) display an unweighted network and its *clique complex*, obtained by promoting cliques to simplices. Simplices can be intuitively thought as higher-dimensional interactions between vertices, e.g. as a simplex the clique (*b,c,i*) corresponds to a filled triangle and not just its sides. The same principle applies to cliques—thus simplices—of higher order. (Online version in colour.)

Homology is a topological invariant that characterizes a topological space *X* by counting its holes and their dimensions. By hole, we mean a hollow region bounded by the parts of that space. The dimension of a hole is directly related to the dimension of its boundary. The boundary of a two-dimensional hole is a one-dimensional loop; the three-dimensional inner part of a doughnut, where the filling goes, is bounded by two-dimensional surface; for dimensions higher than 2, it becomes difficult to have a mental representation of a hole, but *k*-dimensional holes are still bounded by (*k* − 1) dimensional faces. In our work, we start with a network and from it construct a topological space. We now use figure 1 to show how we proceed and make rigorous what we mean by boundaries and holes.

In a network like that of figure 1*a*, we want the ring of nodes (*a*,*b*,*c*,*d*) to be a good candidate for a one-dimensional boundary, whereas the other rings of three nodes should not constitute interesting holes. The reason for this choice comes from the formalization of the notion of hole. One way to formalize this is by opposition that is we define what we mean by a dense subnetwork in order to highlight regions of reduced connectivity, i.e. holes. The most natural and conservative definition we can adopt for a dense subnetwork is that of *clique*, a completely connected subgraph [27]. Moreover, cliques have the crucial property, which will be important later, of being nested, i.e. a clique of dimension *k* (*k*-clique) contains all the *m*-cliques defined by its nodes with *m* < *k*. Using this definition and *filling in* all the maximal cliques, the network in figure 1*a* can be represented as in figure 1*b*: 3-cliques are filled in, becoming tiles, and the only interesting structure left is the square (*a*,*b*,*c*,*d*). It is important at this point to note that a *k*-clique can be seen as a *k* − 1 simplex, i.e. as the convex hull of *k*-points. Our representation of a network can thus be seen as a topological space formed by a finite set of simplices that by construction satisfy the condition that defines the type of topological spaces called abstract simplicial complexes [28]: each element of the space is a simplex, and each of its faces (or subset in the case of cliques) is also a simplex.

This condition is satisfied, because each clique is a simplex, and subsets of cliques are cliques themselves, and the intersection of two cliques is still a clique.

The situation with weighted networks becomes more complicated. In the context of a weighted network, the holes can be thought of as representing regions of reduced connectivity with respect to the surrounding structure.

**Figure 2.**
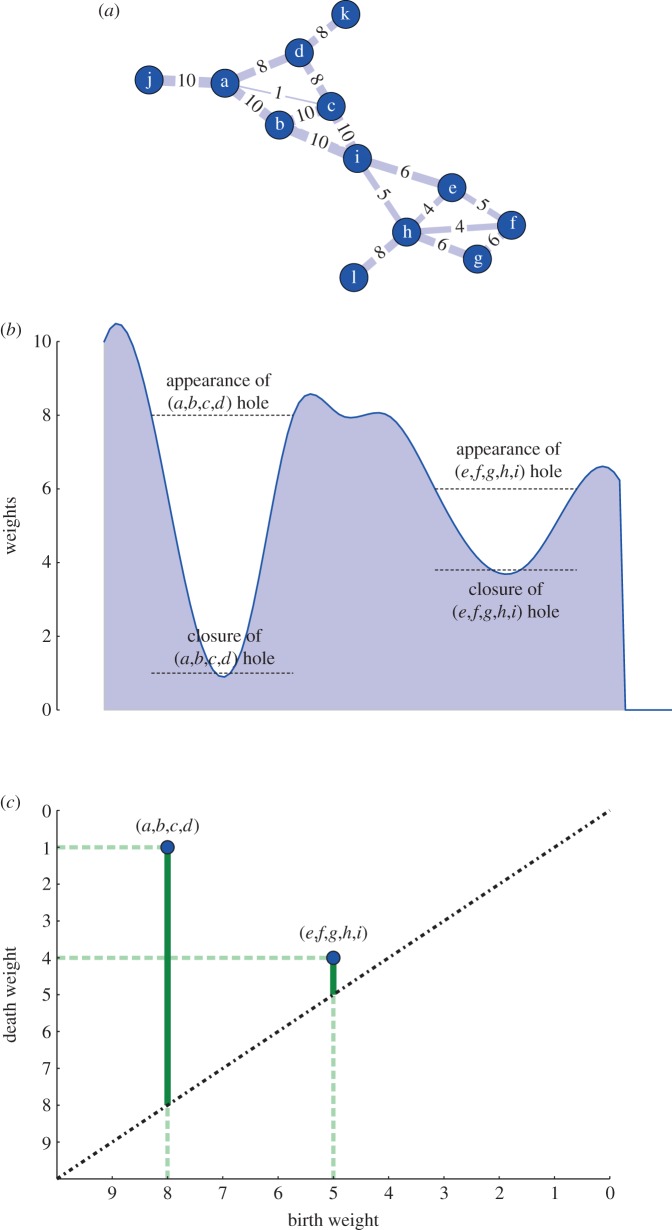
Panels (*a­–c*) display a weighted network (*a*), its intuitive representation in terms of a stratigraphy in the weight structure according the weight filtration described in the main text (*b*) and the persistence diagram for *H*_1_ associated with the network shown (*c*). By promoting cliques to simplices, we identify network connectivity with relations between the vertices defining the simplicial complex. By producing a sequence of networks through the filtration, we can study the emergence and relative significance of specific features along the filtration. In this example, the hole defined by (*a*,*b*,*c*,*d*) has a longer persistence (vertical solid green bars) implying that the boundary of the cycle are much heavier than the internal links that eventually close it. The other hole instead has a much shorter persistence, surviving only for one step and is therefore considered less important in the description of the network homological properties. Note that the births and deaths are defined along the sequence of descending edge weights in the network, not in time. (Online version in colour.)

Consider, for example, the case depicted in figure 2*a*: the network is almost the same as figure 1 with the two exceptions that it now has weighted edges and has an additional very weak edge between nodes *a* and *c*. The edges in the cycle [*a*,*b*,*c*,*d*] are all much stronger than the link (*a*,*c*) that closes the hole by making (*a*,*b*,*d*) and (*b*,*c*,*d*) cliques and therefore fills them. The loop (*e*,*f*,*g*,*h*,*i*) has a similar situation, but the difference in edge weights between the links along the cycle and those crossing, is not as large as in the previous case. It would be therefore useful to be able to generalize the approach exposed earlier for binary networks to the case of weighted networks in such a way as to be able to measure the difference between the two cases (*a*,*b*,*c*,*d*) and (*e*,*f*,*g*,*h*,*i*). As shown by figure 2*b*, this problem can be intuitively thought of as a stratigraphy in the link-weight fabric of the network, where the aim is to detect the holes, measure their depth and when they appear as we scan across the weights' range.

From figure 2*b*, it becomes clear that the added value of this method over conventional network techniques lies in its capability to describe mesoscopic patterns that coexist over different intensity scales, and hence to complement the information about the community structure of brain functional networks. A way to quantify the relevance of holes is given by *persistent homology*. We describe it and its application to the case of weighted networks in full detail in §3.

## 3. A persistent homology of weighted networks

The method that we adopt was introduced in references [29,30] and relies on an extension of the metrical persistent homology theory originally introduced by references [31,32]. Technical details about the theory of persistent homology and how the computation is performed can be found in the works of Carlsson, Zomorodian and Edelsbrunner [28,31–35]. Persistent homology is a recent technique in computational topology developed for shape recognition and the analysis of high dimensional datasets [36,37]. It has been used in very diverse fields, ranging from biology [38,39] and sensor network coverage [40] to cosmology [41]. Similar approaches to brain data [42,43], collaboration data [44] and network structure [45] also exist. The central idea is the construction of a sequence of successive approximations of the original dataset seen as a topological space *X*. This sequence of topological spaces *X*_0_, *X*_1_, … , *X_N_* = *X* is such that 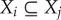 whenever *i* < *j* and is called the *filtration*. Choosing how to construct a filtration from the data is equivalent to choosing the type of goggles one wears to analyse the data.

In our case, we sort the edge weights in descending order and use the ranks as indices for the subspaces. More specifically, denote by 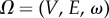 the functional network with vertices *V*, edges *E* and weights 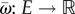. We then consider the family of binary graphs *G_*ω*_* = (*V*, *E_*ω*_*), where an edge *e ∈ E* is also included in *G_*ω*_* if its weight *ω_e_* is larger than *ω* (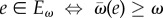).

To each of the *G_*ω*_*, we associate its *clique*, or *flag complex K_*ω*_*, that is the simplicial complex that contains the *k*-simplex [*n*_0_, *n*_1_, *n*_2_, … *n_k_*_− 1_] whenever the nodes *n*_0_, *n*_1_, *n*_2_, … *n_k_*_−1_ define a clique in *G_*ω*_* [27]. As subsets of cliques and intersections of cliques are cliques themselves, as we pointed out in §2, our clique complex is thus a particular case of a simplicial complex.

The family of complexes {*K_ω_*} defines a filtration, because we have 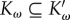 for *ω* > *ω*′. At each step, the simplices in *K_ω_* inherit their configuration from the underlying network structure and, because the filtration swipes across all weight scales in descending order, the holes among these units constitute mesoscopic regions of reduced functional connectivity.

Moreover, this approach also highlights how network properties evolve along the filtration, providing insights about where and when lower connectivity regions emerge. This information is available, because it is possible to keep track of each *k*-dimensional cycle in the homology group *H_k_*. A generator uniquely identifies a hole by its constituting elements at each step of the filtration process. The importance of a hole is encoded in the form of ‘time-stamps' recording its birth *β_g_* and death *δ_g_* along the filtration {*K_ω_*} [31]. These two time-stamps can be combined to define the *persistence π_g_* = *δ_g_* − *β_g_* of a hole, which gives a notion of its importance in terms of it lifespan. Continuing the analogy with stratigraphy, *β_g_* and *δ_g_* correspond, respectively, to the top and the bottom of a hole and *π_g_* would be its depth. As we said above, a generator 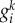, or hole, of the *k*th homology group *H_k_* is identified by its birth and death along the filtration. Therefore, 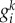 is described by the point 
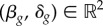
. A standard way to summarize the information about the whole *k*th persistent homology group is then to consider the diagram obtained plotting the points corresponding to the set of generators. The (multi)set {(*β_g_*,*δ_g_*} is called the persistence diagram of *H_k_*. In figure 2*c*, we show the persistence diagram for the network shown in figure 2*a* for *H*_1_. Axes are labelled by weights in decreasing order. It is easy to check that the coordinates correspond exactly to the appearance and disappearance of generators. The green vertical bars highlight the persistence of a generator along the filtration. The further a point is from the diagonal (vertically), the more persistent the generator is. In §4, we introduce two objects, the persistence and the frequency homological scaffolds, designed to summarize the topological information about the system.

## 4. Homological scaffolds

Once one has calculated the generators 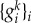 of the *k*th persistent homology group *H_k_*, the corresponding persistence diagram contains a wealth of information that can be used, for example, to highlight differences between two datasets. It would be instructive to obtain a synthetic description of the uncovered topological features in order to interpret the observed differences in terms of the microscopic components, at least for low dimensions *k*. Here, we present a scheme to obtain such a description by using the information associated with the generators during the filtration process. As each generator, 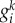 is associated with a whole equivalence class, rather than to a single chain of simplices, we need to choose a representative for each class, we use the representative that is returned by the *javaplex* implementation [46] of the persistent homology algorithm [47]. For the sake of simplicity in the following, we use the same symbol 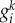 to refer to a generator and its representative cycle.

We exploit this to define two new objects, the *persistence* and the *frequency homological scaffolds* 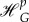 and 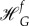 of a graph *G*. The *persistence homological scaffold* is the network composed of all the cycle paths corresponding to generators weighted by their persistence. If an edge *e* belongs to multiple cycles *g*_0_,*g*_1_, … ,*g_s_*, its weight is defined as the sum of the generators' persistence:4.1
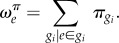


Similarly, we define the *frequency homological scaffold* 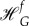 as the network composed of all the cycle paths corresponding to generators, where this time, an edge *e* is weighted by the number of different cycles it belongs to4.2
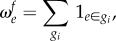


where 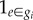 is the indicator function for the set of edges composing *g_i_*. By definition, the two scaffolds have the same edge set, although differently weighted.

The construction of these two scaffolds therefore highlights the role of links which are part of many and/or long persistence cycles, isolating the different roles of edges within the functional connectivity network. The persistence scaffolds encodes the overall persistence of a link through the filtration process: the weight in the persistence scaffold of a link belonging to a certain set of generators is equal to the sum of the persistence of those cycles. The frequency scaffold instead highlights the number of cycles to which a link belongs, thus giving another measure of the importance of that edge during the filtration. The combined information given by the two scaffolds then enables us to decipher the nature of the role different links have regarding the homological properties of the system. A large total persistence for a link in the persistence scaffold implies that the local structure around that link is very weak when compared with the weight of the link, highlighting the link as a locally strong bridge. We remark that the definition of scaffolds we gave depends on the choice of a specific basis of the homology group, and the choice of a consistent basis is an open problem in itself, therefore the scaffolds are not topological invariants. Moreover, it is possible for an edge to be added to a cycle shortly after the cycle's birth in such a way that it creates a triangle with the two edges composing the cycle. In this way, the new edge would be part of the shortest cycle, but the scaffold persistence value would be misattributed to the two other edges. This can be checked, for example, by monitoring the clustering coefficient of the cycle's subgraph as edges are added to it. We checked for this effect and found that in over 80% of the cases the edges do not create triangles that would imply the error, but instead new cycles are created, whose contribution to the scaffold is then accounted for by the new cycle. Finally, we note also that, when a new triangle inside the cycle is created, the two choices of generator differ for a path through a third strongly connected node, owing to the properties of boundary operators. Despite this ambiguity, we show in the following that they can be useful to gain an understanding of what the topological differences detected by the persistent homology actually mean in terms of the system under study.

## 5. Results from fMRI networks

**Figure 3.**
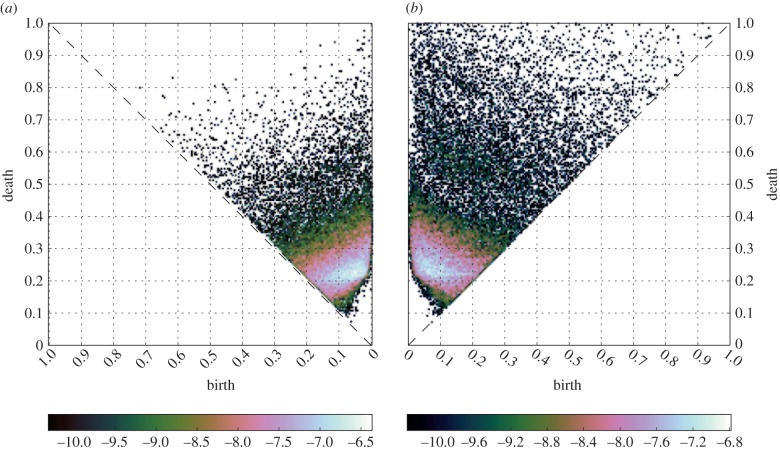
Probability densities for the *H*_1_ generators. Panel (*a*) reports the (log-)probability density for the placebo group, whereas panel (*b*) refers to the psilocybin group. The placebo displays a uniform broad distribution of values for the births–deaths of *H*_1_ generators, whereas the plot for the psilocybin condition is very peaked at small values with a fatter tail. These heterogeneities are evident also in the persistence distribution and find explanation in the different functional integration schemes in placebo and drugged brains. (Online version in colour.)

We start from the processed fMRI time series (see Methods for details). The linear correlations between regional time series were calculated after covarying out the variance owing to all other regions and the residual motion variance represented by the 24 rigid motion parameters obtained from the pre-processing, yielding a partial-correlation matrix *χ^α^* for each subject. The matrices *χ^α^* were then analysed with the algorithm described in the previous sections. We calculated the generators 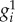 of the first homological group *H*_1_ along the filtration. As mentioned before, each of these generators identifies a lack of mesoscopic connectivity in the form of a one-dimensional cycle and can be represented in a persistence diagram. We aggregate together the persistence diagrams of subjects belonging to each group and compute an associated persistence probability density (figure 3). These probability density functions constitute the statistical signature of the groups' *H*_1_ features.

We find that, although the number of cycles in the groups are comparable, the two probability densities strongly differ (Kolmogorov–Smirnov statistics: 0.22, *p*-value less than 10^−10^).

**Figure 4.**
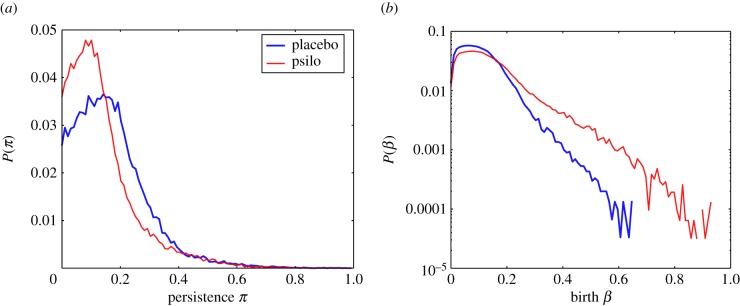
Comparison of persistence *π* and birth *β* distributions. Panel (*a*) reports the *H*_1_ generators' persistence distributions for the placebo group (blue line) and psilocybin group (red line). Panel (*b*) reports the distributions of births with the same colour scheme. It is very easy to see that the generators in the psilocybin condition have persistence peaked at shorter values and a wider range of birth times when compared with the placebo condition. (Online version in colour.)

**Figure 5.**
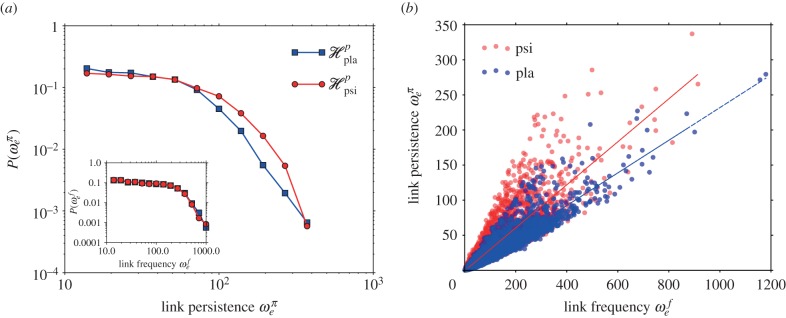
Statistical features of group homological scaffolds. Panel (*a*) reports the (log-binned) probability distributions for the edge weights in the persistence homological scaffolds (main plot) and the frequency homological scaffolds (inset). While the weights in the frequency scaffold are not significantly different, the weight distributions for the persistence scaffold display clearly a broader tail. Panel (*b*) shows instead the scatter plot of the edge frequency versus total persistence. In both cases, there is a clear linear relationship between the two, with a large slope in the psilocybin case. Moreover, the psilocybin scaffold has a larger spread in the frequency and total persistence of individual edges, hinting to a different local functional structure within the functional network of the drugged brains. (Online version in colour.)

The placebo group displays generators appearing and persisting over a limited interval of the filtration. On the contrary, most of the generators for the psilocybin group are situated in a well-defined peak at small birth indices, indicating a shorter average cycle persistence. However, the psilocybin distribution is also endowed with a longer tail implying the existence of a few cycles that are longer-lived compared with the placebo condition and that influences the weight distribution of the psilocybin persistence scaffold. The difference in behaviour of the two groups is made explicit when looking at the probability distribution functions for the persistence and the birth of generators (figure 4), which are both found to be significantly different (Kolmogorov–Smirnov statistics: 0.13, *p*-value < 10^−30^ for persistence and Kolmogorov–Smirnov statistics: 0.14, *p*-value < 10^−35^ for births). In order to better interpret and understand the differences between the two groups, we use the two secondary networks described in §4, 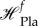 and 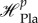 for the placebo group and 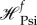 and 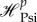 for the psilocybin group. The weight of the edges in these secondary networks is proportional to the total number of cycles an edge is part of, and the total persistence of those cycles, respectively. They complement the information given by the persistence density distribution, where the focus is on the entire cycle's behaviour, with information on single links. In fact, individual edges belonging to many and long persistence cycles represent functionally stable ‘hub’ links. As with the persistence density distribution, the scaffolds are obtained at a group level by aggregating the information about all subjects in each group. These networks are slightly sparser than the original complete *χ^α^* networks5.1
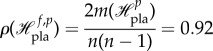
and5.2
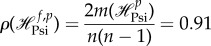
and have comparable densities. A first difference between the two groups becomes evident when we look at the distributions for the edge weights (figure 5*a*). In particular, the weights of 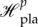 display a cut-off for large weights, whereas the weights of 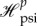 have a broader tail (Kolmogorov–Smirnov statistics: 0.06, *p*-value < 10^−20^; figure 5*a*). Interestingly, the frequency scaffold weights probability density functions cannot be distinguished from each other figure 5*a* (inset) (Kolmogorov–Smirnov statistics: 0.008, *p*-value = 0.72). Taken together, these two results imply that while edges statistically belong to the same number of cycles, in the psilocybin scaffold, there exist very strong, persistent links.

The difference between the two sets of homological scaffolds for the two groups becomes even more evident when one compares the weights between the frequency and persistence scaffolds of the same group. Figure 5*b* is a scatter plot of between the weights of edges from both scaffolds for the two groups. The placebo group has a linear relationship between the two quantities meaning that edges that are persistent also belongs to many cycles (*R*^2^ = 0.95, slope = 0.23). Although the linear relationship is still a reasonable fit for the psilocybin group (*R*^2^ = 0.9, slope = 0.3), the data in this case display a larger dispersion. In particular, it shows that edges in 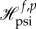 can be much more persistent/longer-lived than in 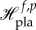 but still appear in the same number of cycles, i.e. the frequency of a link is not predictive of its persistence or simply put: some connections are much more persistent in the psychedelic state. Moreover, the slopes of linear fits of the two clouds are statistically different (*p*-value < 10*^−^*^20^, *n*_pla_ = 13 200 and *n*_psi_ = 13 275 [48]) pointing to a starkly different local functional structure in the two conditions.

The results from the persistent homology analysis and the insights provided by the homological scaffolds imply that although the mesoscopic structures, i.e. cycles, in the psilocybin condition are less stable than in the placebo group, their constituent edges are more stable.

## 6. Discussion

In this paper, we first described a variation of persistent homology that allows us to deal with weighted and signed networks. We then introduced two new objects, the homological scaffolds, to go beyond the picture given by persistent homology to represent and summarize information about individual links. The homological scaffolds represent a new measure of topological importance of edges in the original system in terms of how frequently they are part of the generators of the persistent homology groups and how persistent are the generators to which they belong to. We applied this method to an fMRI dataset comprising a group of subjects injected with a placebo and another injected with psilocybin.

By focusing on the second homology group *H*_1_, we found that the stability of mesoscopic association cycles is reduced by the action of psilocybin, as shown by the difference in the probability density function of the generators of *H*_1_ (figure 3).

**Figure 6.**
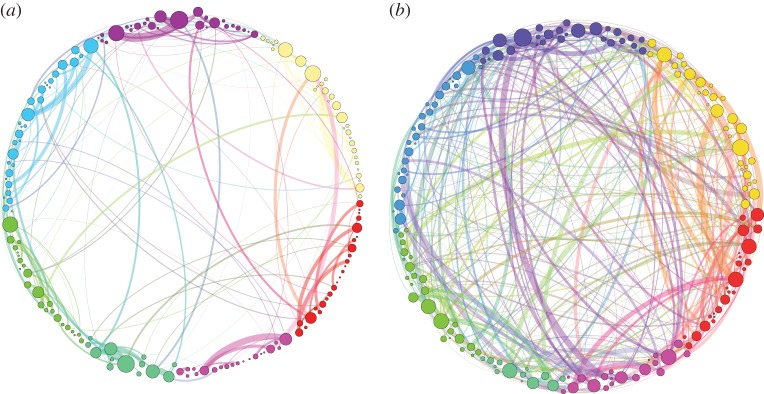
Simplified visualization of the persistence homological scaffolds. The persistence homological scaffolds 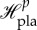 (*a*) and 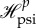 (*b*) are shown for comparison. For ease of visualization, only the links heavier than 80 (the weight at which the distributions in figure 5*a* bifurcate) are shown. This value is slightly smaller than the bifurcation point of the weights distributions in figure 5*a*. In both networks, colours represent communities obtained by modularity [49] optimization on the placebo persistence scaffold using the Louvain method [50] and are used to show the departure of the psilocybin connectivity structure from the placebo baseline. The width of the links is proportional to their weight and the size of the nodes is proportional to their strength. Note that the proportion of heavy links between communities is much higher (and very different) in the psilocybin group, suggesting greater integration. A labelled version of the two scaffolds is available as GEXF graph files as the electronic supplementary material. (Online version in colour.)

It is here that the importance of the insight given by the homological scaffolds in the persistent homology procedure becomes apparent. A simple reading of this result would be that the effect of psilocybin is to relax the constraints on brain function, ascribing cognition a more flexible quality, but when looking at the edge level, the picture becomes more complex. The analysis of the homological scaffolds reveals the existence of a set of edges that are predominant in terms of their persistence although they are statistically part of the same number of cycles in the two conditions (figure 5). In other words, these functional connections support cycles that are especially stable and are only present in the psychedelic state. This further implies that the brain does not simply become a random system after psilocybin injection, but instead retains some organizational features, albeit different from the normal state, as suggested by the first part of the analysis. Further work is required to identify the exact functional significance of these edges. Nonetheless, it is interesting to look at the community structure of the persistence homological scaffolds in figure 6. The two pictures are simplified cartoons of the placebo (figure 6*a*) and psilocybin (figure 6*b*) scaffolds. In figure 6*a,b*, the nodes are organized and coloured according to their community membership in the placebo scaffold (obtained with the Louvain algorithm for maximal modularity and resolution 1 [50]). This is done in order to highlight the striking difference in connectivity structure in the two cases. When considering the edges in the tail of the distribution, weight greater than or equal to 80, in figure 5*a*, only 29 of the 374 edges present in the truncated psilocybin scaffold are shared with the truncated placebo scaffold (165 edges). Of these 374 edges, 217 are between placebo communities and are observed to mostly connect cortical regions. This supports our idea that psilocybin disrupts the normal organization of the brain with the emergence of strong, topologically long-range functional connections that are not present in a normal state.

The two key results of the analysis of the homological scaffolds can therefore be summarized as follows (i) there is an increased integration between cortical regions in the psilocybin state and (ii) this integration is supported by a persistent scaffold of a set of edges that support cross modular connectivity probably as a result of the stimulation of the 5HT2A receptors in the cortex [51].

We can speculate on the implications of such an organization. One possible by-product of this greater communication across the whole brain is the phenomenon of synaesthesia which is often reported in conjunction with the psychedelic state. Synaesthesia is described as an inducer-concurrent pairing, where the inducer could be a grapheme or a visual stimulus that generates a secondary sensory output—like a colour for example. Drug-induced synaesthesia often leads to chain of associations, pointing to dynamic causes rather than fixed structural ones as may be the case for acquired synaesthesia [52]. Broadly consistent with this, it has been reported that subjects under the influence of psilocybin have objectively worse colour perception performance despite subjectively intensified colour experience [53].

To summarize, we presented a new method to analyse fully connected, weighted and signed networks and applied it to a unique fMRI dataset of subjects under the influence of mushrooms. We find that the psychedelic state is associated with a less constrained and more intercommunicative mode of brain function, which is consistent with descriptions of the nature of consciousness in the psychedelic state.

## 7. Methods

### 7.1. Dataset

A pharmacological MRI dataset of 15 healthy controls was used for a proof-of-principle test of the methodology [54]. Each subject was scanned on two separate occasions, 14 days apart. Each scan consisted of a structural MRI image (T1-weighted), followed by a 12 min eyes-close resting-state blood oxygen-level-dependent (BOLD) fMRI scan which lasted for 12 min. Placebo (10 ml saline, intravenous injection) was given on one occasion and psilocybin (2 mg dissolved in 10 ml saline) on the other. Injections were given manually by a study doctor situated within the scanning suite. Injections began exactly 6 min after the start of the 12-min scans, and continued for 60 s.

#### 7.1.1. Scanning parameters

The BOLD fMRI data were acquired using standard gradient-echo EPI sequences, reported in detail in reference [54]. The volume repetition time was 3000 ms, resulting in a total of 240 volumes acquired during each 12 min resting-state scan (120 pre- and 120 post-injection of placebo/psilocybin).

#### 7.1.2. Image pre-processing

fMRI images were corrected for subject motion within individual resting-state acquisitions, by registering all volumes of the functional data to the middle volume of the acquisition using the FMRIB linear registration motion correction tool, generating a six-dimension parameter time course [55]. Recent work demonstrates that the six parameter motion model is insufficient to correct for motion-induced artefact within functional data, instead a Volterra expansion of these parameters to form a 24 parameter model is favoured as a trade-off between artefact correction and lost degrees of freedom as a result of regressing motion away from functional time courses [56]. fMRI data were pre-processed according to standard protocols using a high-pass filter with a cut-off of 300 s.

Structural MRI images were segmented into *n* = 194 cortical and subcortical regions, including white matter cerebrospinal fluid (CSF) compartments, using Freesurfer (http://surfer.nmr.mgh.harvard.edu/), according to the Destrieux anatomical atlas [57]. In order to extract mean-functional time courses from the BOLD fMRI, segmented T1 images were registered to the middle volume of the motion-corrected fMRI data, using boundary-based registration [58], once in functional space mean time-courses were extracted for each of the *n* = 194 regions in native fMRI space.

#### 7.1.3. Functional connectivity

For each of the 194 regions, alongside the 24 parameter motion model time courses, partial correlations were calculated between all couples of time courses (*i*,*j*), non-neural time courses (CSF, white matter and motion) were discarded from the resulting functional connectivity matrices, resulting in a 169 region cortical/subcortical functional connectivity corrected for motion and additional non-neural signals (white matter/CSF).

### 7.2. Persistent homology computation

For each subject in the two groups, we have a set of persistence diagrams relative to the persistent homology groups *H_n_*. In this paper, we use the *H*_1_ persistence diagrams of each group to construct the corresponding persistence probability densities for *H*_1_ cycles.

Filtrations were obtained from the raw partial-correlation matrices through the Python package *Holes* and fed to *javaplex* [46] via a Jython subroutine in order to extract the persistence intervals and the representative cycles. The details of the implementation can be found in reference [30], and the software is available at Holes [59].

## Supplementary Material

Psilocybin homological scaffold

## Supplementary Material

Placebo homological scaffold
